# Neural dynamics of personality trait perception and interaction preferences

**DOI:** 10.1038/s41598-024-76423-9

**Published:** 2024-12-12

**Authors:** Martin Weiß, Marko Paelecke, Patrick Mussel, Grit Hein

**Affiliations:** 1 Center of Mental Health, Department of Psychiatry, Psychosomatic and Psychotherapy, Translational Social Neuroscience Unit, University Hospital Würzburg, Margarete-Höppel-Platz 1, 97080 Würzburg, Germany; 2 Department of Psychology I: Clinical Psychology and Psychotherapy, Institute of Psychology, University of Würzburg, Würzburg, Germany; 3 Department of Psychology V: Differential Psychology, Personality Psychology and Psychological Diagnostics, Institute of Psychology, University of Würzburg, Würzburg, Germany; 4 Division for Psychological Diagnostics and Differential Psychology, Psychologische Hochschule Berlin, Berlin, Germany

**Keywords:** Social neuroscience, Human behaviour

## Abstract

According to recent research, self-reported Big Five personality traits are associated with preferences for faces that are representative of certain Big Five traits. Previous research has primarily focused on either preference for distinct prototypical personality faces or the accuracy of trait ratings for these faces. However, the underlying neural correlates involved in the processing of prototypical personality faces are unknown. In the present study, we aim to bridge this gap by investigating whether participants’ Big Five personality traits predict preferences to interact with individuals represented by prototypical personality faces, as well as the neural processing of these facial features. Based on theoretical considerations and previous research, we focus on trait extraversion, agreeableness and neuroticism, and corresponding prototypical faces. Participants were asked to classify prototypical faces as above or below average representative of a certain trait, and then provide an interaction preference rating while face-sensitive event-related potentials (N170 and late positive potential) were measured. In line with our hypotheses, the results showed an interaction preference for faces that were perceived as high (vs. low) extraverted and agreeable and low (vs. high) neurotic. In addition, the preference for agreeable faces interacted with personality characteristics of the perceiver: The higher a persons’ score on trait agreeableness, the higher the face preference ratings for both prototypical and perceived high agreeable faces. Analyses of ERP data showed that an increase in preference ratings for prototypical agreeable faces was paralleled by an increase of the late positive potential. Notably, the N170 did not show any neural signature of the hypothesized effects of personality faces. Together, these results highlight the importance of considering both perceiver characteristics as well as perceived features of an interaction partner when it comes to preference for social interaction.

*Protocol registration* The stage 1 protocol for this Registered Report was accepted in principle on the 8th of May 2023. The protocol, as accepted by the journal, can be found at: 10.17605/OSF.IO/G8SCY.

## Introduction

Facial features have a crucial function in social interactions as they enable perceivers to quickly infer information about the other person^1,2^. Humans are able to accurately infer the personality of their interaction partners beyond chance based on their facial features^3–6^. These impressions correlate, for example, to judgments about the trustworthiness, competence, dominance, and likability of their counterpart^7–9^. A growing body of research shows that independent observers commonly agree on a person’s personality traits based solely on an image of their face^9–11^. Real-life correlates of inferred personality traits from faces include voting decisions^12^, leadership potential^13^ and sentencing decisions^14^. Consequently, there is evidence for preferences for distinct personality traits inferred from faces^3^. One method to create faces that represent distinct personality characteristics is based on so-called “prototype” faces, which are used to extract the defining facial features of a group of persons, thereby removing the features that make each face appear individual^15^. In the current research, we are interested in whether participants’ Big Five personality traits interact with the identification of personality traits of prototypical faces ^4^ and how they correlate with the neural processing of others’ faces. We aim to elucidate whether the preference to interact with distinct persons is shaped by the perceiver’s personality and their neural processing of the face. To narrow down the hypothesis space in the current study and informed by literature^15–17^, we focus on participants’ and the other person’s personality regarding trait extraversion (E), agreeableness (A), and neuroticism (N).

An important aspect is whether similarity or dissimilarity is the driver for the preference to interact with a person. The literature shows that similarity is more important at zero acquaintance than in long-term relationships^18^. Accordingly, personality similarity predicts satisfaction with college roommates^19^. More specifically, social interaction quality is rated higher by participants when partners are similarly extraverted whereas similarly disagreeable partners exhibit poor interaction quality^16^. However, high personality similarity can also have a negative effect on spouse satisfaction, and these negative effects are particularly pronounced when the partners are similarly extraverted and conscientious^20^. When persons are asked about ideal romantic partners, both men and women prefer someone who is more conscientious, extraverted, and agreeable, but less neurotic than themselves^17^.

### Insights from studies using prototypical personality faces

Research on the relationship between facial features and personality is ongoing, and the findings are not always consistent. We present a brief overview of the research regarding personality faces of extraversion, agreeableness, and neuroticism.

#### Extraversion

Features describing individuals who are high in extraversion include talkativeness, sociability, dominance, assertiveness, excitement seeking, self-confidence, and positive emotions^21^. Borkenau and colleagues^22^ showed that even after 50 ms, their participants were able to accurately infer strangers’ extraversion from their faces. Prototypical faces of highly extraverted persons are generally preferred^23^. Moreover, Brown and colleagues^3^ investigated whether social exclusion increases preferences for faces representing high levels of extraversion. They found that only men showed increased preferences for extraverted faces after experiencing social exclusion.

#### Agreeableness

Individuals high in agreeableness are characterized by the tendency to be cooperative, considerate, kind, empathetic and willing to compromise with others^24^. Similar to extraversion, agreeableness can be accurately inferred from faces^25^ and individuals generally prefer faces with high over low agreeableness^23^. Concerning interactions between participant trait and protype faces, women with high levels of trait neuroticism prefer male faces representing high levels of agreeableness^23^. Scholars argue that highly agreeable male faces might represent an increased probability of marital satisfaction^26^. In line with this reasoning, Brown and colleagues showed that women with restricted sociosexual attitudes (i.e., preference for long-lasting relationships) tended to prefer agreeable male faces^27^.

#### Neuroticism

High levels of neuroticism are characterized by the tendency to experience negative emotions such as anxiety, fear, and sadness. Individuals who score high in neuroticism tend to be more emotionally reactive and may be more prone to experiencing negative emotions in response to stress or other challenges^28,29^. Women with higher levels of neuroticism prefer faces of less neurotic men^23^, which might indicate a preference for emotionally stable partners^30^. However, these women also prefer faces of highly neurotic women, potentially signaling approach to similar conspecifics^23^.

In summary, past research identified relationships between participants’ characteristics and preferences for prototypical faces of extraversion, agreeableness, and neuroticism. However, most of the studies investigated generic preference based on a forced-choice paradigm where participants indicated their preference for faces representing high or low levels of the same trait^3,23,27^, and focused on specific motives for these preferences (e.g., disease concerns)^31^. Therefore, more refined associations between participants’ personality and their preferences for social interactions with persons represented by high and low personality trait faces have not yet been established.

### Event-related potentials

To date, no study has yet examined the neural processing of prototypical personality faces, i.e., faces that are highly or lowly representative of a particular trait. To bridge this gap in research, we used event-related potentials (ERPs) that indicate early and late stages of the processing of prototypical personality faces. The N170 brain potential is a face-sensitive component of neural processing ^32^ that is characterized by a negative deflection that is largest over the lateral temporo-occipital brain areas with a peak latency of around 170 ms after stimulus onset^33–35^. Research has shown correlations between N170 amplitudes and the fusiform face area^36^, the occipital face area^37^, and the superior temporal sulcus^38^, reflecting face-specific structural encoding^35,39^. With regard to the present research, there is evidence suggesting that the N170 may be involved in the processing of subtle differences in personality traits as depicted in faces. Dzhelyova and colleagues reported an increased negativity of N170 amplitudes in response to untrustworthy-looking male faces but also to trustworthy-looking female faces^40^. Other studies showed a smaller N170 amplitude for highly attractive and averagely attractive faces compared to less attractive faces^41^. Mechanistically, these results support the idea that distinct levels of processing (i.e., differences in N170 amplitudes) may reflect the configural similarity of other people’s faces to facial prototypes developed through a lifetime of experience with faces^41^.

In contrast, the late positive potential (LPP) is a positive deflection that is typically largest in the centroparietal regions of the brain and has a peak latency that typically begins 300 ms after stimulus onset^42^. The LPP indicates processes of stimulus evaluation and controlled attention^43–45^, and relates to cortical and subcortical brain areas associated with emotional processing (e.g., amygdala, ventral striatum)^46,47^. However, research has shown that personal closeness modulates the neural processing when viewing faces as the LPP is increased in response to one’s own face^48^, faces of romantic partners^49^, and faces of relatives^50^. Therefore, – inter alia – Hajcak and Foti^42^ propose that LPP reflects stimulus significance rather than the mere sensitivity to emotional stimuli. Translated to the present research, Hajcak’s and Foti’s^42^ framework supports the assumption that (dis-)similar prototypical personality faces might influence their neural processing.

While most studies investigating face processing have used faces expressing different valence (i.e., smiling, angry and sad faces)^51–54^ there is evidence that ERPs in response to neutral faces can be influenced by contextual information such as personal relevance^55–57^. Because valence crowds out more subtle nuances in facial features (e.g.^58^), we used neutral faces to examine the interplay between personality traits of the perceiver and the inferred personality traits of the other person when predicting neural responses to their faces. Importantly, there is a lack of research that explicitly focuses on neutral faces. In studies that test valence effects, neutral faces typically serve as control condition (e.g.^51,52,54^).

Individual differences in personality have been shown to correlate with face-related LPP amplitudes across a variety of traits, e.g., agreeableness^59^, empathy^60^, anxiety^61–63^, depression^64,65^, or schizotypy^66^. For instance, Krasowski and colleagues studied the influence of framing with negative or neutral biographical information on neural correlates.^59^ The authors could show that low trait agreeableness was associated with increased processing of potentially hostile faces. Yet, evidence on the influence of broad personality traits in terms of Big Five and the processing of prototypical personality faces is still missing. Although different kinds of prototypical personality faces have been used a lot in behavioral studies^3–6^, there is no investigation on their neural underpinnings.

Here, we investigated how individual differences in Big Five personality traits affect preferences for interaction with other persons represented by prototypical personality faces and their neural processing. For this purpose, participants were presented with faces that are either prototypical of high or low levels of a trait. Subsequently, participants were asked to classify these faces as being representative of high versus low levels of a given trait. This response, in relation to the actual prototypicality of the face, quantified the accuracy with which participants estimate personality traits from faces. Next, participants answered a preference rating in which they were asked how much they would like to interact with this person. Specifically, we tested our hypotheses (see below) separately based on the prototypicality of faces, and based on participants’ subjective perception of these faces, distinguishing between objective and subjective representativeness of these faces and their impact on participants’ preference ratings and neural processing.

### Manipulation check

To test the validity of the prototypical faces, we assumed that participants would be more accurate than chance in classifying the other person’s facial features according to their prototypicality on a distinct trait ^3–5^. 

#### Hypotheses

Each of the following hypotheses were tested twice, i.e., one model was performed with prototypical personality (highly vs. lowly representative) as a predictor, and another model was performed using perceived representativeness (below-average or above-average representative) as a predictor. In theory, the effects should be comparable, which is why we do not formulate separate hypotheses for the two different methodological approaches. If results diverge between prototypical personality models and perceived representativeness models, this could be an indicator that there are interindividual differences in the perception of prototypical personality faces, and that mental representations in subjective perception could be more important than objective features of the stimuli. For an overview of the hypotheses, see Table 1.

**Table 1:** Design table.

Question	Hypothesis (if applicable)	Sampling plan (e.g., power analysis)	Analysis plan	Interpretation given to different outcomes
Do individuals generally prefer to interacting with extraverted, agreeable and emotionally stable persons?	H1: Participants prefer persons with facial features representing high levels of E (H1a) or A (H1b), and low levels of N (H1c)	We conducted a safeguard power analysis^70^ using the lower boundary of the two-tailed 60% confidence interval for an assumed effect size of *d* = 0.62 based on pilot data on ratings of representativeness for agreeableness faces. As the Cohen’s d for the N170 and LPP were expected to be larger based on previous research (d = 2.5 and d = 1.15, respectively; for details, see Method section), we based the sample size calculation on *d* = 0.62. The safeguard analysis resulted in an estimated sample size of *N* = 82 assuming α = 0.05 and power = 1-β = 0.80	For the following models, we compared a model with an intercept per participant with a model with an intercept per participant and face, as well as models with and without random slopes for the main fixed effects and select the model with the better fit **Linear mixed models** (exemplary random intercept models) prototypical personality models: preference rating ~ facial features *(E + A + N) + (1|participant) and perceived representativeness models: preference rating ~ response*(E + A + N) + (1|participant)	*Significant main effect of facial features/response:* A. participants report higher (E and A)/ lower (N) preferences for highly prototypical/ perceived representative faces (E and A and N) compared to lowly representative faces. This result would confirm H1a-H1c B. participants report lower (E and A)/ higher (N) preferences for highly prototypical/ perceived representative faces (E and A and N) compared to lowly representative faces. This result would contradict H1a-H1c *No significant main effects of facial features /response:* This result would contradict H1a-H1c
Is there an interaction between an individual’s personality (E, A and N) and their preference to interact with extraverted, agreeable and emotionally stable persons?	H2: Higher preference for faces representing a) E for participants with higher levels of E (H2a), b) A for participants with higher levels of A (H2b) and c) N for participants with higher levels N (H2c), respectively			*Significant interactions between facial features /response and trait (E, A, or N):* A. participants show higher preferences for E (H2a), A (H2b), and N (H2c) faces, when they report high levels of E (H2a), A (H2b), and N (H2c) themselves, respectively. This result would confirm H2a-H2c B. participants show lower preferences for E (H2a), A (H2b), and N (H2c) faces, when they report high levels of E (H2a), A (H2b), and N (H2c) themselves, respectively. This result would contradict H2a-2c *No significant interactions between facial features /response and trait (E, A, or N):* This result would contradict H2a-H2c
Are there different neural signatures for the prototypical faces of extraverted, agreeable, and emotionally stable persons with regard to N170 and LPP?	H3: N170 amplitudes are larger for faces representing high levels of E (H3a) and A (H3b) and low levels of N (H3c) compared to faces representing low levels of E or A and high levels of N H4: LPP amplitudes are larger for faces representing high levels of E (H4a) and A (H4b) and low levels of N (H4c) compared to faces representing low levels of E or A and high levels of N		**Linear mixed models** (exemplary random intercept models) prototypical personality models: N170/LPP ~ facial features *(E + A + N) + (1|participant) and perceived representativeness models: N170/LPP ~ response*(E + A + N) + (1|participant)	*Significant main effect of facial features /response:* A. participants show higher (E and A)/ lower (N) N170/LPP amplitudes for highly prototypical/ perceived representative faces (E and A and N) compared to lowly representative faces. This result would confirm H3a-4c and H4a-4c B. participants show lower (E and A)/ higher (N) N170/LPP amplitudes for highly prototypical/ perceived representative faces (E and A and N) compared to lowly representative faces. This result would contradict H3a-3c and H4a-H4c *No significant main effects of facial features /response:* This result would contradict H3a-H3c and H4a-H4c
Is there an interaction between an individual’s personality (E, A and N) and the neural processing of prototypical faces of extraverted, agreeable and emotionally stable persons with regard to N170 and LPP?	H5: Larger N170 for faces representing a) E for participants with higher levels of E (H5a), b) A for participants with higher levels of A (H5b) and c) N for participants with higher levels N (H5c), respectively H6: Larger LPP for faces representing a) E for participants with higher levels of E (H5a), b) A for participants with higher levels of A (H5b) and c) N for participants with higher levels N (H5c), respectively			*Significant interactions between facial features /response and trait (E, A, or N):* A. participants show higher N170/LPP amplitudes for E (H5a/H6a), A (H5b/H6b), and N (H5c/H6c) faces, when they report high levels of E (H5a/H6a), A (H5b/H6b), and N (H5c/H6c) themselves, respectively. This result would confirm H5a-H5c and H6a-H6c B. participants show lower N170/LPP amplitudes for E (H5a/H6a), A (H5b/H6b), and N (H5c/H6c) faces, when they report high levels of E (H5a/H6a), A (H5b/H6b), and N (H5c/H6c) themselves, respectively. This result would confirm H5a-H5c and H6a-H6c *No significant interactions between facial features /response and trait (E, A, or N):* This result would contradict H5a-H5c and H6a-H6c
Are there brain-behavior relationships between N170 brain potentials and the preference to interact with another person?	H7: N170 amplitudes (E: H7a, A: H7b, N: H7c) predict preference ratings		**Linear mixed models** (exemplary random intercept models) prototypical personality model: preference rating ~ N170/LPP* facial features *(E + A + N) + (1|participant) and perceived representativeness model: preference rating ~ N170/LPP*response*(E + A + N) + (1|participant)	*Significant main effect of N170 amplitude:* A. participants with higher N170 amplitudes show higher preference ratings. This result would confirm H7a-H7c B. participants with lower N170 amplitudes show higher preference ratings. This result would contradict H7a-H7c *No significant main effects of N170 amplitude:* This result would contradict H7a-H7c
Are there brain-behavior relations between LPPs and the preference to interact with another person?	H8: LPP amplitudes (E: H8a, A: H8b, N: H8c) predict preference ratings			*Significant main effect of LPP:* A. participants with higher LPP amplitudes show higher preference ratings. This result would confirm H8a-H8c B. participants with lower LPP amplitudes show higher preference ratings. This result would contradict H8a-H8c *No significant main effects of LPP:* This result would contradict H8a-H8c

A = Agreeableness, E = Extraversion, N = Neuroticism, LPP = Late Positive Potential

Regarding preference ratings, we expected that participants prefer interacting with persons showing facial morphology representing/being perceived as prototypical for high levels of extraversion (H1a) and agreeableness (H1b) and low levels of neuroticism (H1c), independent of the participants’ own personality ^17^. In line with research on similarity effects at zero acquaintance, we expected a) participants with higher levels of trait extraversion would show higher preference for faces representing/being perceived as prototypical for high levels of extraversion (H2a), b) participants with higher levels of agreeableness would show higher preference for faces representing/being perceived as prototypical for high levels of agreeableness (H2b) and c) participants with higher levels of neuroticism would show higher preference for faces representing/being perceived as prototypical for high levels of neuroticism (H2c)^17,18^.

At the neural level, for both N170 and the early, middle, and late parts of the LPP, we expected that the main effects (H3a-H3c and H4a-H4c) and interaction effects (H5a-H5c and H6a-H6c) for preference ratings would be reflected by the corresponding increase in amplitudes of ERPs, reflecting the neural processes underlying the preference and similarity effect. In line with this reasoning, we expected amplitudes of the N170 (H7a-H7c), and early, middle and late part of the LPP (H8a-H8c) to predict preferences ratings. A possible functional account for why the N170 and LPP may respond differently to faces varying in extraversion, agreeableness, and neuroticism is that these traits may be associated with different patterns of neural activity during face processing. Specifically, individual differences in traits related to social cognition and emotional processing may impact the N170 component by modulating the processing of social cues, such as facial features. For example, individuals high in extraversion may exhibit increased N170 amplitude due to their current social attitude towards other people or particularly salient cues. On the other hand, individual differences in traits related to emotional processing and regulation, such as neuroticism or agreeableness, may impact the LPP component by modulating the allocation of attentional resources, the evaluation of emotional significance, and the regulation of emotional responses to faces^67–69^.

## Method

### Sample

Our pilot data showed an effect size of ${\eta }_{p}^{2}$ = 0.09 (= Cohen’s d of 0.62) for *facial features* on representativeness ratings for agreeableness faces. As there were no data on the neural correlates of prototypical personality faces, we based the sample size calculation for the neural correlates on evidence from studies using emotional faces and more subtle facial features (e.g., trustworthiness, dominance) as an indicator of which effects in the modulation of neural correlates are expected. This approach resulted in an average Cohen’s d of 2 for the N170, i.e., average of studies showing ${\upeta }_{\text{p}}^{2}$ = 0.60^71^, ${\upeta }_{\text{p}}^{2}$ = 0.54^72^, ${\upeta }_{\text{p}}^{2}$ = 0.70^73^, and ${\upeta }_{\text{p}}^{2}$ = 0.16^74^. For the LPP, we used an average Cohen’s d of 1.09 (${\upeta }_{\text{p}}^{2}$= 0.24^73^, ${\upeta }_{\text{p}}^{2}$ = 0.26^75^, and ${\upeta }_{\text{p}}^{2}$ = 0.20^76^). In summary, d = 0.62 for the subjective representativeness ratings was the smallest effect size of interest. Thus, as a conservative estimate, we based our power analysis on the hypothesis with the smallest effect size. We conducted a safeguard power analysis^70^ as we wanted to account for potentially lower effect sizes for interaction terms between participant trait and facial features. To account for this uncertainty, we used the lower boundary of the two-tailed 60% confidence interval for an assumed effect size of *d* = 0.62, thereby increasing the probability of accurately determining the population effect size. The result indicated a required sample size of *N* = 82, assuming α = 0.05 and power = 1−β = 0.8. We recruited participants via an online recruitment portal of the University of Würzburg and they received a compensation of 15€ for their participation. We invited participants with normal or corrected-to-normal vision, and only those who indicated by self-report that they had neither suffered from any psychiatric or neurological disease within the past 10 years, nor did they take any psychoactive drugs or medication. The study was carried out in accordance with the recommendations of “Ethical guidelines, The Association of German Professional Psychologists” (“Berufsethische Richtlinien, Berufsverband Deutscher Psychologinnen und Psychologen”). All participants gave written informed consent in accordance with the Declaration of Helsinki before they participated in the experiment. The protocol was approved by the ethics committee of the University Hospital of Würzburg.

### Task and procedure

Personality traits were assessed at the beginning of the laboratory session. First, participants answered the IPIP-NEO-120 personality questionnaire^77^ as we were interested in their broad Big-Five personality factors and a depression inventory (PHQ-8^78^) for exploratory purposes.

**Fig. 1:**
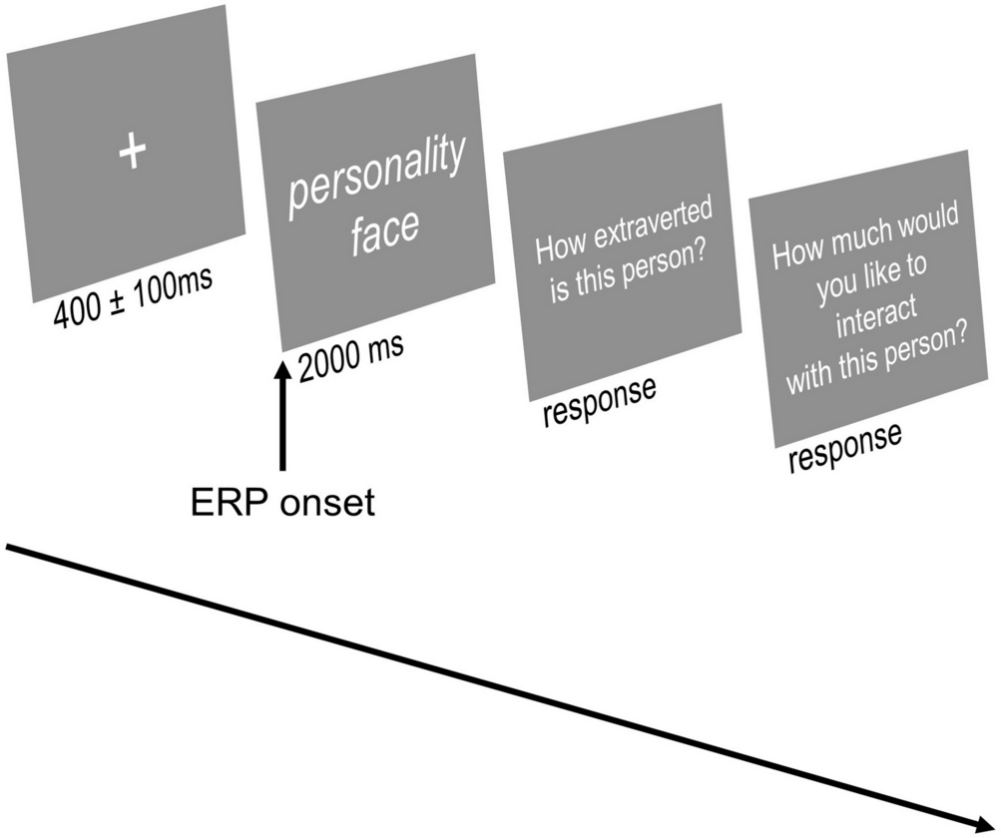
Example trial procedure. At the beginning of each block, participants were instructed to focus on a specific personality trait, followed by a description of characteristics describing a person as above- or below-average on the trait of interest in this block.

Afterwards, participants sat down on a chair in a sound-attenuated, dimly lit testing room and provided informed consent before EEG electrodes were applied. Afterwards, participants performed a “personality quiz”. We presented three trait blocks in randomized order targeting E, A, and N, respectively. In each block, participants evaluated faces high or low on a given trait (E, A, or N). At the beginning of each block, participants were instructed to focus on a specific trait for the upcoming trials (e.g., “please consider how extraverted you think the following persons are”) followed by a description of characteristics describing a person as above-average or below-average on the trait of interest in this block. Each trial (see Fig. 1) started with a fixation cross (400 ± 100 ms) and the presentation of a photograph of a face (for details, see Stimulus Material) for 2 s. Participants were asked to rate the personality of the face (trait rating: e.g., “how extraverted is this person?”) using a binary answering format (below-average vs. above-average). The left and right arrow key corresponded to high and low respectively for half the participants, and vice versa for the other half of the participants, and were assigned in a randomized fashion. In addition, participants indicated on a 9-point likert scale how much they would like to interact with that person (preference rating; 1 = “not at all”, 9 = “very much”). There were 80 trials per block based on sex (two levels: male vs. female), facial features (two levels: highly vs. lowly representative), and 20 different faces for each condition (see Stimulus material). As we treated sex as a methodological component and therefore aim to ensure a balanced representation of male and female faces in both experimental conditions to minimize any potential confounds, there were 40 faces (i.e., 20 male and 20 female faces) for each level of facial features (i.e., 40 high representative faces and 40 low representative faces). Thus, there were 3 × 80 = 240 trials in total. The whole experiment took approx. 35 min.

### Stimulus material

**Fig. 2:**
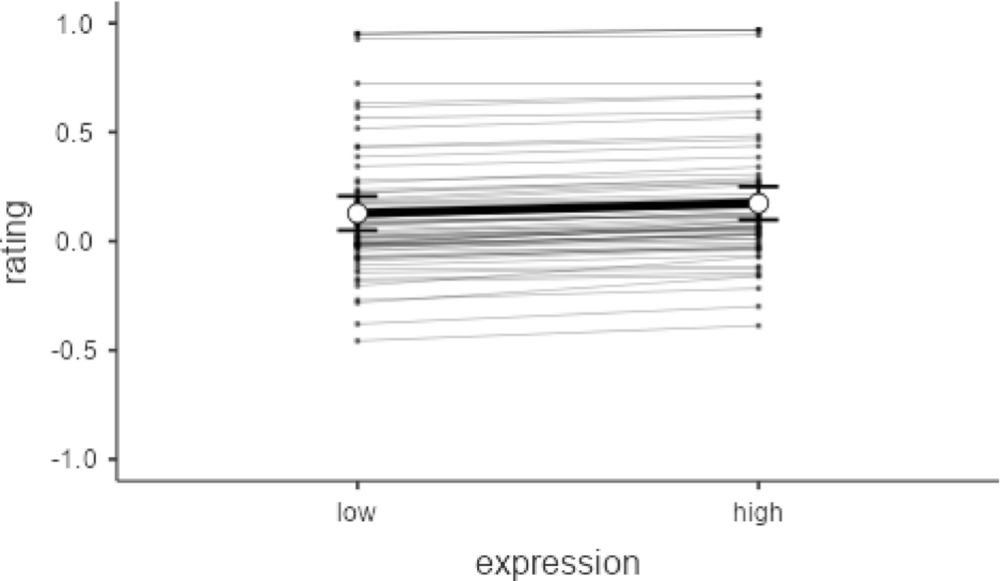
Results of the pilot study on agreeableness faces (N = 69). Participants rated faces as either above-average (1), average (0) or below-average (−1). Results of a linear mixed model using facial features as predictor for the rating indicated that prototypical high agreeableness faces are rated as significantly more representative compared to low faces (*p* = 0.013). Random effects are plotted per participant.

As facial stimuli for this paradigm, we used a set of images from a previous study^23^. This set includes 20 male and 20 female Caucasian face identities from the Aging Faces^79^ and Chicago Face Databases^80^, which were chosen to represent potential interaction partners. Each of these 40 faces was morphed with a composite face prototypical of high or low levels of each Big Five trait, respectively^4^. This procedure resulted in 40 composites of male and female faces representing high self-reported levels of each trait and 40 composites of male and female faces representing low self-reported levels of each trait. Thus, each original neutral face identity was presented twice, once as a 70/30 blend (i.e., 70% prototype face/30% original face) with a face that is prototypical of high levels of a trait and once as a 70/30 blend with a face prototypical of low levels of a trait. Faces were presented in a random order, except that the same face was never repeated in two consecutive trials. We tested the applicability of these blends in a pilot study using agreeableness faces (see Fig. 2). The results showed that participants rated faces representing high A as being more representative compared to low A faces (β = 0.05, *t*(1, 68) = 2.56, p = 0.013). In the pilot study, we used a three-level answering format (below-average, average, above-average). However, we omitted the “average” option to facilitate answering and adapt the format to the analysis for the current study (see Statistical Analyses).

### EEG measurement and analysis

EEG was recorded using a 32-channel system (ActiCap; Brain Products, Munich, Germany) based on active Ag/AgCl electrodes, placed according to the 10–20 system. Electrophysiological activity was recorded via Brain Vision Recorder (Brain Products GmbH, München, Germany) and a 32-channel DC amplifier system using BrainAmp Amplifier software (Brain Products GmbH, München, Germany). During recording, impedances were kept below 10 kΩ and electrodes were referenced to FCz. For further computation, MATLAB and EEGLAB toolbox^81^ were used as well as a variant of the EPOS pipeline^82^. First, data were re-referenced to the average across scalp electrodes and electrode FCz were reinstated. Then, artefact-laden channels were automatically rejected based on their averaged *z*-score (*z*-value threshold 3.29) according to the dimensions kurtosis, probability, and spectrum. The segmentation of the data was applied 1000 ms before an event to 2000 ms after the event. Next, the data was high-pass filtered with a cut-off frequency of 1 Hz to get a more stable independent component analysis (ICA) solution^83^. A first ICA was performed for trial rejection and trials exceeding the criterion of z = 3.29 for joint probability or kurtosis. A second ICA was performed on the remaining trials^84^, removing all components associated with muscular activity or eye movement and blink activity by using the program SASICA^85^ for selection with input from ADJUST^86^ and MARA^87^. The ICA solution was written to the unfiltered data. Data was baseline-corrected (baseline from − 200 ms to 0 ms, i.e., the event onset) and excluded channels were interpolated. Finally, data was transformed to current source density using a script provided by Cohen^88^ to reduce the impact of volume conduction on the recorded potentials, and data was filtered with a 0.1 Hz high-pass filter and a 30-Hz low-pass filter^71,72^.

N170 peak latency was defined as the maximal negative peak between 125 to 220 ms at pooled electrode sites P7/P8^89,90^. Individual N170 amplitudes per condition were scored as averages in the area ± 20 ms around the respective grand average peaks^54,91^. For the quantification of the LPP, we analyzed three separate time windows from 400–600 ms, 600–800 ms, and 800–1000 ms for early, middle, and late portions of the LPP component^92^. To account for the broad distribution of the LPP, we scored mean activity in the respective time window at centro-parietal electrode sites CP1, Cz, CP2, P3, Pz, and P4^72,93–95^.

### Data exclusion

We replaced participants who consistently rated all faces as either low or high in at least one trait block as this would indicate that they did not differentiate at all between the faces, as well as participants who showed no variance in the preference ratings (indicating the same preference to interact with all individuals). To ensure that our data were of sufficient quality for EEG analysis, we only included participants who had a minimum of 28 valid trials in each experimental condition (i.e., 28 valid trials of lowly and 28 valid trials of highly representative faces for each personality trait). This threshold was based on recommendations from Jensen and MacDonald^96^ for the minimum number of valid trials needed for reliable N170 brain potentials. Participants who did not meet this criterion would be replaced.

### Statistical analyses

All statistical analyses were performed at the single trial level with linear mixed-effects models and generalized mixed-effects models (for binomial variables; i.e. Mixed Effects Logistic Regressions) in R-Studio 2022.12.0 on *R* version 4.2.2 and the packages “lme4” version 1.1–31^97^ and “ggplot2” version 3.4.0^98^. For each of the following analyses, a separate model was computed for each set of prototypical personality faces (i.e., E, A, and N faces). We applied simple contrast coding for two-levelled predictors (i.e., −1 and 1) to test whether the effect of one level of the predictor on the dependent variable was significantly different from the mean of the other level of the predictor^99^. Following each analysis, we compared the fit of a model including a random intercept per participant to a model including random intercepts per participant and per face. Random slopes were included in the analysis to improve the accuracy of the models and reduce the risk of Type 1 errors^100^. The models with and without random slopes were compared to verify that there were no significant differences between the models. Using the *anova* function, we conducted a Likelihood Ratio Test and used the model that provided the significantly better fit. In case of a non-significant model comparison, we selected the model with the lower Akaike information criterion (AIC). The manipulation check, i.e., the accuracy of participants’ personality judgement of each face, was analyzed with a generalized mixed model and the binary outcome variable correct (true vs. false) in each block. We included *facial features* (highly vs. lowly representative) as predictor. The preference to interact with a certain person (H1 and H2) was analyzed using linear mixed models with the preference rating as metric outcome variable. For the models using the prototypicality of the faces, we entered *facial features* (highly vs. lowly representative), standardized scores of participants’ E, A, and N, and the two-way interactions between *facial features* and each participant trait as predictors. For the models using the perceived representativeness of the faces, we entered *response* (i.e., whether participants rate the faces as above- or below-average representative), standardized scores of participants’ E, A, and N, and the two-way interactions between *response* and each participant trait as predictors.

Similarly, we analyzed the N170 (H3 and H5) and the LPP (H4 and H6) in response to each set of prototypical faces with a linear mixed model. For the models using the prototypicality of the faces, we entered *facial features* (highly vs. lowly representative), standardized scores of participants’ E, A, and N, and the two-way interactions between *facial features* and each participant trait as predictors. For the models using the perceived representativeness of the faces, we entered *response* (i.e., whether participants rate the faces as above- or below-average representative), standardized scores of participants’ E, A, and N, and the two-way interactions between *response* and each participant trait as predictors.

Finally, to test for brain-behavior relations (H7 and H8), we extended the models for H1 and H2 by adding standardized amplitudes of N170 or LPP, respectively. For the models using prototypicality of faces, we included three-way interactions between standardized N170/LPP, *facial features* (highly vs. lowly representative), and standardized scores for each participant trait. For the models using the perceived representativeness of the faces, we allowed for three-way interactions between standardized N170/LPP, *response* (i.e., whether participants rate the faces as above- or below-average representative) and standardized scores of each participant trait.

We originally had planned to perform further exploratory analyses to investigate whether the interactions between participant gender and the sex of the personality faces have moderating effects on the confirmatory analyses. However, due to the strong gender imbalance of participants (79% women), we refrained from conducting these analyses in order to avoid drawing any biased conclusions.

To facilitate the readability of the results section, we only report significant confirmatory effects. The complete results including not hypothesized results of the preferred models according to AIC comparison are reported in detail in the Supplement.

## Results

### Sample

Data collection took place between August and November 2023. 92 participants took part in the study. Four participants were excluded due to technical problems, one participant aborted the study, and two participants were excluded because they rated all faces as either low or high in at least one trait block. The average number of interpolated channels was 2.8 (SD = 1.16). After rejection of artifact-laden trials, the average number of trials included in the analyses for the high and low prototypical faces separately for female and male faces per trait block was 19.7 (SD = 0.60, range 17–20), so no participant had to be replaced (minimum 28 trials required across both sexes). Finally, we analyzed 85 individuals (mean age = 26.22, SD = 8.84; 67 women [79%]).

### Manipulation check

Prototypical high agreeableness faces were identified with higher accuracy (59.4%) compared to low agreeableness faces (42.7%; Odds Ratio [OR] = 1.4, 95% confidence interval [CI] = [1.33; 1.47], *p* < 0.001; see Table 2). In the extraversion (OR = 0.93; 95% CI = [0.89; 0.98], *p* = 0.004) and the neuroticism block (OR = 0.76, 95% CI = [0.72; 0.80], *p* < 0.001), faces with a prototypical low facial features of the trait (extraversion: 52.4%; neuroticism: 56.7%) were more accurately identified compared to faces with high prototypical facial features (extraversion: 48.8%; neuroticism: 42.9%). All accuracies > 50% (low extraversion, high agreeableness, and low neuroticism faces) significantly surpassed chance level in a one-sided *t-*test indicating more correct than incorrect classification for these faces (all *t*(84) ≥ 1.82, all values of *p* ≤ 0.036). The trait questionnaires showed good reliability (Cronbach’s α = 0.86—0.87; see Table 2).

**Table 2:** Descriptive statistics on the accuracy of the prototypical personality faces in the personality quiz, the preference ratings and the trait questionnaires.

	**Extraversion faces**		**Agreeableness faces**		**Neuroticism faces**	
	**High**	**Low**	**High**	**Low**	**High**	**Low**
Accuracy [%]	48.82	52.35	59.40	42.66	42.88	56.72
Preference rating [mean ± SE] for *prototypical* personality	4.65 ± 0.03	4.58 ± 0.03	5.03 ± 0.03	4.97 ± 0.03	5.01 ± 0.03	4.84 ± 0.03
Preference rating [mean ± SE] for *perceived* personality	5.00 ± 0.02	4.25 ± 0.03	5.75 ± 0.02	3.94 ± 0.03	4.70 ± 0.03	5.09 ± 0.03

	**Mean ± SE**	**Min**	**Max**	**Min (observed)**	**Max (observed)**	**Cronbach’s α**
Trait extraversion	82.11 ± 1.32	24	120	51	115	0.86
Trait agreeableness	95.25 ± 1.11	24	120	60	111	0.86
Trait neuroticism	59.38 ± 1.38	24	120	33	104	0.87

### Preference ratings

**Fig. 3:**
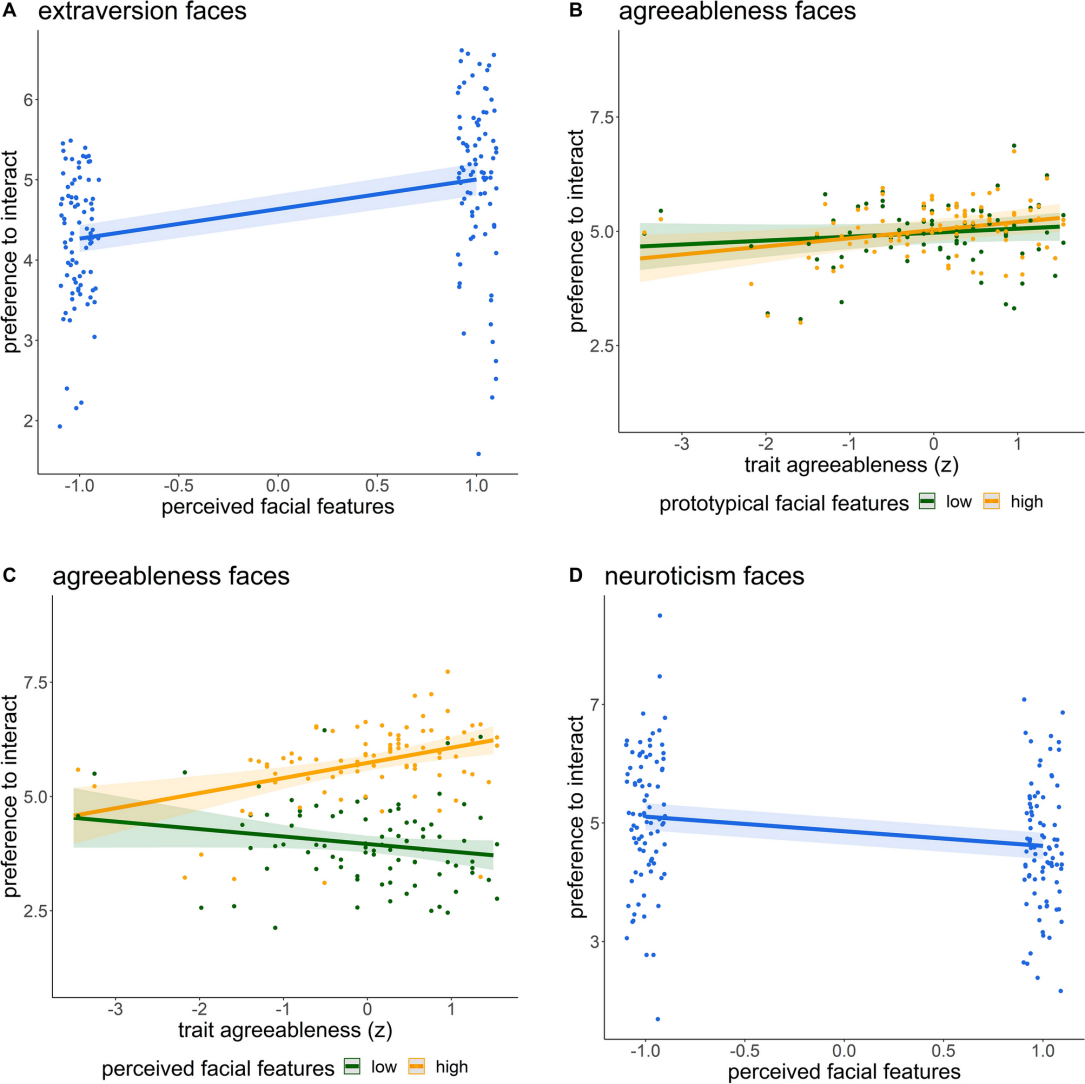
Hypothesized results of the preference ratings. **A**. Faces perceived as highly extraverted were preferred (compared to low perceived extraversion). **B**. Self-reported agreeableness predicts increasing preference for prototypical high (vs low) agreeableness faces. **C**. Self-reported agreeableness predicts increased preference for faces perceived as high (vs. low) agreeable **D.** Faces perceived as highly neurotic were less preferred (compared to low perceived neuroticism). Shaded areas represent the 95% confidence interval.

Faces perceived as highly extraverted were associated with higher preference ratings (*B* = 0.37, CI = [0.26; 0.47], *p* < 0.001; Fig. 3A). Self-reported trait extraversion scores predicted general preference across all faces in the extraversion block (prototypical facial features model:* B* = 0.19, CI = [0.01; 0.37], *p* = 0.038; perceived facial features model: *B* = 0.21, CI = [0.03; 0.39], *p* = 0.020).

Prototypical high agreeableness faces lead to higher preference ratings for persons with higher trait levels of agreeableness (*B* = 0.05, CI = [0.01; 0.08], *p* = 0.009; Fig. 3B). Faces perceived as highly agreeable were generally preferred (*B* = 0.89, CI = [0.75; 1.02], *p* < 0.001). Self-reported trait agreeableness predicted general preference across prototypical agreeableness faces (*B* = 0.13, CI = [0.01; 0.26], *p* = 0.039). There was an interaction of perceived agreeableness and self-reported agreeableness. The effect of perceived facial features on interaction preference was higher for participants scoring high on trait agreeableness (*B* = 0.25, CI = [0.11; 0.38], *p* < 0.001; Fig. 3C).

In the neuroticism face block, faces perceived as highly neurotic faces were rated lower in the preference (*B* = −0.25, CI = [-0.43; −0.07], *p* = 0.008; Fig. 3D).

### Neural activation

**Fig. 4:**
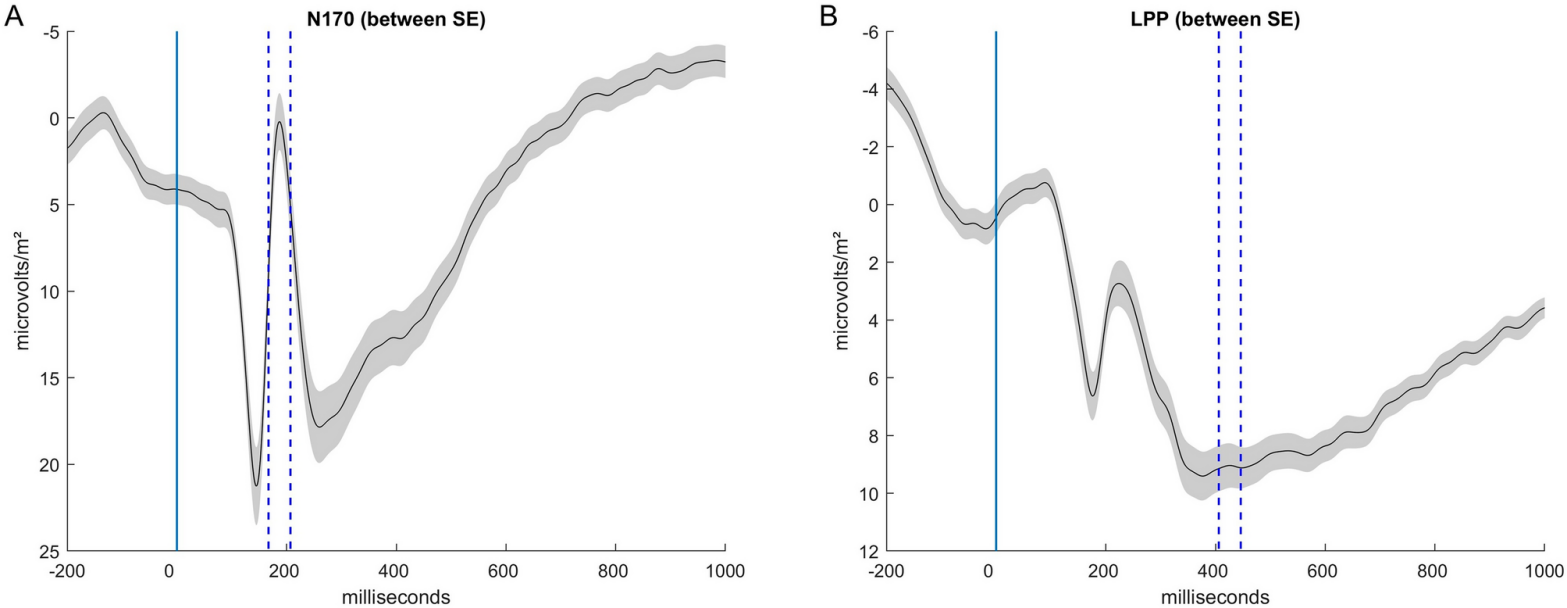
Detection of peak values for the analysis of the N170 (electrode sites P7 and P8, Panel **A**) and the late positive potential (LPP; electrode sites CP1, Cz, CP2, P3, Pz, and P4; Panel** B**). For the LPP, we have only indicated the early part, as it represents the most positive part of the LPP.

In all models on the N170 and the early parts of the LPP, none of the hypothesized effects showed a significant result. See Fig. 4, for peak detection time-windows of the N170 and the (early) LPP.

**Fig. 5:**
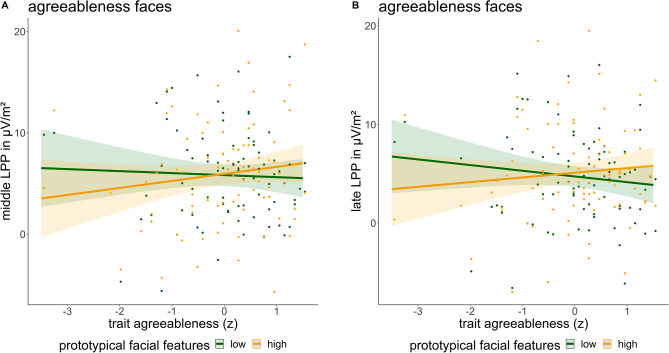
Interaction between trait agreeableness and prototypical facial features of agreeableness faces in predicting middle (**A**) and late (**B**) positive potential amplitudes. Shaded areas represent the 95% confidence interval.

Concerning the middle and late parts of the LPP, we found an interaction between self-reported agreeableness and high vs. low prototypical agreeableness faces, in that for individuals scoring high on trait agreeableness prototypical high agreeableness faces lead to higher middle LPP amplitudes (*B* = 0.45, CI = [0.04; 0.85], *p* = 0.032, Fig. 5A) as well as higher late LPP amplitudes (*B* = 0.53, CI = [0.11; 0.94], *p* = 0.013; Fig. 5B).

### Brain-behavior relations

We tested whether the significant and/or hypothesized effects observed in ratings are modulated by neural activity. A comprehensive overview of the brain-behavior relations can be found in the Supplement (Tables S 18–29).

**Fig. 6:**
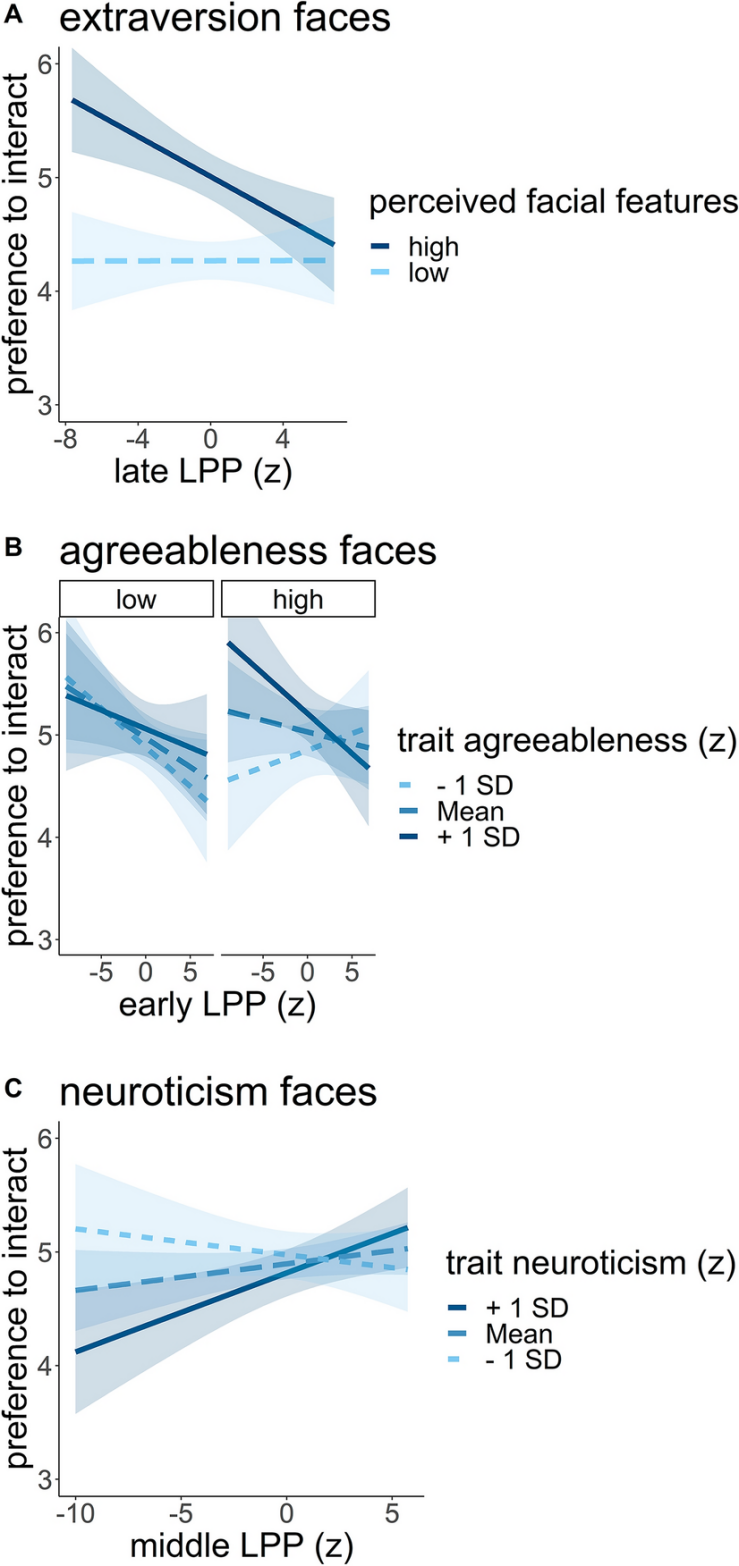
Results of brain-behavior analyses. **A** Interaction between perceived facial features and late part LPPs in the prediction of preferences regarding extraversion faces. **B** Interaction between trait agreeableness, early LPPs and prototypical agreeableness faces. **C** Interaction between the middle LPP and trait neuroticism regarding prototypical neuroticism faces. Shaded areas represent the 95% confidence interval.

The main effect of perceived facial features regarding extraversion faces, was moderated by the middle (*B* = −0.04, CI = [−0.08; 0.00], *p* = 0.050) and late LPP (*B* = −0.04, CI = [−0.08; −0.01], *p* = 0.020, Fig. 6A). Higher LPP for faces perceived as high (vs. low) extraverted resulted in lower preference ratings.

The interaction between trait agreeableness and prototypical agreeableness faces was moderated by individual differences in the early part of the LPP (*B* = −0.03, CI = [-0.07; 0.00], *p* = 0.046). Simple slopes analyses revealed that with increasing early LPPs prototypical high agreeableness faces were less preferred for individuals with high trait agreeableness (*p* = 0.04) and that prototypical low agreeableness faces were less preferred with increasing early LPPs and low (*p* = 0.05) or average trait agreeableness (*p* = 0.03; Fig. 6B).

Although not significant in the behavioral model, we could show a moderation of the middle LPP on self-reported neuroticism in the neuroticism face block for the prototypical and the perceived model (prototypical facial features model: *B* = 0.05, CI = [0.01; 0.09], *p* = 0.027, Fig. 6C; perceived facial features model: *B* = 0.05, CI = [0.01; 0.09], *p* = 0.021). Simple slopes analyses showed that for individuals with high trait neuroticism, increasing levels of middle LPP were associated with higher preference ratings (*p* = 0.01).

## Discussion

In this study, we investigated whether and how one’s personality and the prototypical as well as the perceived personality of faces jointly influence the preference for interacting with that person. The results showed that perceived personality influences this preference, as perceived high agreeableness and high extraversion had a positive effect on preference ratings, whereas perceived high neuroticism was detrimental. In terms of self-reported and perceived personality interactions, higher prototypical and perceived agreeableness is preferred by agreeable participants. In ERPs, prototypical highly agreeable faces were associated with higher late LPPs in highly agreeable participants. While expected effects on the N170 were completely absent, there was a complex pattern of LPP-related brain-behavior relations which, however, did not coherently reflect the aforementioned results.

### Extraversion faces

First of all, results were in line with the hypothesis that perceived high extraversion faces is linked to increased preference ratings – although this effect was absent for the prototypicality of these faces. Recent research showed that extraverted individuals prefer to interact in their daily lives with others they perceive as extraverted^101^ and that dyads of two extraverts or two introverts had better initial interactions compared to dyads with one extravert and one introvert^16^. Regarding brain-behavior relations, we could demonstrate a significant modulation of the reported effect by neural activation. With increasing amplitude of the LPP faces perceived as highly extraverted, the preference rating decreased. From the perspective of cognitive resource allocation, a stronger neural response could increase the likelihood that a stimulus might be less preferred due to its effortful processing^102^. In the present study, we did not find a trait interaction between participants’ extraversion and the prototypical or perceived personality facial features of the faces. We did find, however, an unexpected interaction between participant neuroticism and the perceived personality facial features, which has – as far as we know – not been documented in the literature so far. One speculative explanation could be that people with a higher level of neuroticism perceive less extraverted faces as less pleasant, as a high level of extraversion in another person could overwhelm them.

### Agreeableness faces

According to our results, agreeable persons prefer hypothetical social interactions with *objectively* and *subjectively* likeminded people – that is, with those who (on average) describe themselves and whom they perceive as kind, sympathetic, cooperative, warm, frank, and considerate as themselves. In a recent study on interpersonal dynamics during a conversation in previously-unfamiliar dyads, agreeable dyads reported more positive affect^103^. This personality-congruent selection effect^104,105^ is in line with theories proposing that individuals prefer to interact with similar others (e.g., similarity-attraction theory, social identity theory, and self-categorization theory)^106–108^. The increase in LPP amplitudes for prototypical high agreeableness faces in highly agreeable persons is supported by the conceptualization of LPP as an indicator of stimulus significance^42^. Thus, results were in line with all our hypotheses on prototypical/perceived agreeableness faces, the moderation by trait agreeableness as well as corresponding neural activation, although only in one of four components (late LPP). Regarding brain-behavior relations, agreeable individuals with low early LPPs showed an increase in preference ratings for prototypical high agreeableness faces (Fig. 6B). Research on facial attractiveness showed that a weaker face encoding in an earlier component was related to adopting a majority vote of face attractiveness instead of focusing on the actual features of a face^109^. Thus, in our study, weaker encoding of prototypical faces with high agreeableness could promote a focus on some core features of agreeableness, increasing preference for these faces.

### Neuroticism faces

Our results showed that highly neurotic faces are associated with lower preference ratings, which fits well with the literature showing that individuals prefer less neurotic interaction partners^17,101^. In the current study, we could not show significant neural correlates of neuroticism faces, as other research on the impact of trait neuroticism on neural signatures of emotional image processing^110^. However, we found that participants with high trait neuroticism who also had a higher middle LPP showed higher overall preference ratings for faces in the neuroticism block, suggesting that the effect patterns for revealing neuroticism-related neural face processing may be complex.

Extending our hypotheses and although not specific to high neuroticism faces, agreeable individuals showed a general preference for all faces in the neuroticism face block. This is consistent with our recent findings that agreeable individuals prefer everyday interactions with people they perceive as having higher neuroticism^101^. Moreover, agreeable individuals showed an increase in preference ratings for all faces in the neuroticism face block at all levels of later LPPs, but most strongly at low to average LPPs. One speculative explanation would be that agreeable persons who rather superficially process features of both low and high neuroticism faces prefer interactions with such individuals as they might detect more automatically that these individuals offer more room for their own personality to unfold.

### Prototypicality vs. perception of personality faces

In our study, we found that perceived personality had a stronger influence on preference ratings for extraversion and neuroticism than prototypical personality traits, which played a greater role for agreeableness. The fact that some of the hypothesized effects were confirmed for these very subtle cues (i.e. all faces show neutral emotional expressions) suggests that the immediate visual and emotional information in a person’s face play a crucial role in how they are socially evaluated. Perceived personality is dynamic and context-dependent and is often shaped by first impressions and social interactions, whereas prototypical personality is more abstract and less influenced by situational factors^111,112^. The larger effects observed at the perceptual level emphasize the importance of subjective interpretations in social preferences and interactions and highlight the nuanced and complex nature of human social cognition.

### Absence of effects on the N170

The lack of significant results for the N170 may be attributed to the specific nature of the N170 and the focus of our research. The N170 is an ERP component that is sensitive to the structural encoding of faces and is typically activated by early perceptual processes involving facial features such as the eyes and overall facial structure^113^. In our study on the personality traits extraversion, agreeableness, and neuroticism, higher-order cognitive and emotional processes were involved that likely extend beyond the early perceptual domain captured by the N170. Furthermore, as discussed in a review^114^, less frequent emotional modulation of the N170 component was documented during active task performance, suggesting that the "personality quiz” in our study may have elicited competing processes involved in attention and emotion decoding at this stage of processing. This task on these personality traits might therefore require more complex cognitive evaluations that are reflected in later ERP components such as the LPP^102^.

### Limitations and outlook

We would like to highlight some limitations of our study. First, a more gender-balanced sample would have been preferable to generalize the results across genders or to identify robust gender differences. Second, we observed some residual activity in the baseline of our ERPs (see Fig. 4; for a more detailed illustration per trait and facial features, see Figure S2). This could be attributed to a stimulus-preceding negativity^115^, as the fixation cross was only jittered by ± 100 ms, potentially leading participants to anticipate the photograph. Such stimulus-preceding negativity (e.g., Bereitschaftspotential or readiness potential) occurs approx. 1 s before the stimulus (or reaction). Another possibility is that this residual activity represents an overlapping process from the previous response. To shorten the task duration, the fixation cross was presented immediately after the response, and our stimulus was introduced 300-500 ms after the preference rating, which might not allow sufficient time for the processing of the preference rating. Future studies should include a larger inter-trial interval to overcome this limitation. Third, apart from the agreeableness faces, the results for the prototypical personality and the perceived personality of the faces did not coincide. Besides conceptual differences between prototypical and perceived differences, as discussed above, the largely absent effects on prototypical faces could call into question the validity of the personality faces used in the study (which was also the debate of a recent review by Bovet and colleagues)^116^. For personality researchers, a cross-validated face database (e.g. self- and other-ratings of personality by known and strangers) with different individuals representing the same trait would be beneficial in order to avoid undesirable effects of morphing (e.g. different morphing strategies can lead to different perceptions of the stimulus)^117^.

## Conclusion

In the current study we aimed to systematically investigate whether and how Big Five personality traits interact with the preference to interact with individuals represented by prototypical personality faces, as well as the neural processing of these faces. With regard to agreeableness, we identified a similarity effect – agreeable individuals would prefer interactions with persons represented by prototypical and perceived highly agreeable faces. On a neural level, this effect is supported by increased late positive potentials for agreeable persons looking at highly prototypical agreeableness faces. While faces perceived as high in extraversion resulted in increased social preference, those perceived as high in neuroticism were less preferred. Further research is needed to validate the neural processing and brain-behavior relations of prototypical faces and to embed them in the context of social decisions.

## Data Availability

The data that support the findings of this study and the scripts to reproduce the analyses are available on the Open Science Framework at https://osf.io/g8scy/.
